# Basal *PIR* expression in HeLa cells is driven by NRF2 via evolutionary conserved antioxidant response element

**DOI:** 10.1007/s11010-013-1931-0

**Published:** 2014-01-05

**Authors:** Kamil Brzóska, Tomasz M. Stępkowski, Marcin Kruszewski

**Affiliations:** 1 Centre for Radiobiology and Biological Dosimetry, Institute of Nuclear Chemistry and Technology, Dorodna 16, 03-195 Warsaw, Poland; 2 Independent Laboratory of Molecular Biology, Institute of Rural Health, Jaczewskiego 2, 20-090 Lublin, Poland

**Keywords:** Pirin, PIR, NF-κB, NRF2, NFE2L2, ARE

## Abstract

Pirin, a product of the *PIR* gene, is an iron-binding protein acting as a transcriptional coregulator implicated in the regulation of the NF-κB-related transcription via interaction with RelA (p65), as well as BCL3 and NF-κB1 (p50) proteins. Alterations in pirin expression were observed in various tumors and under oxidative stress conditions. The aim of the present work was to analyze the regulation of the transcription of the human *PIR* gene. Using constructs containing a different sized *PIR* promoter and the luciferase reporter genes we found that in HeLa cells *PIR* transcription is mostly dependent on a highly conserved antioxidant response element located 281 bp downstream of the transcription start site. We have proved that the NRF2 transcription factor binds to this element in vivo and drives the basal *PIR* expression. We hypothesize that regulation of the *PIR* expression may constitute a mechanism by which NRF2 is able to modulate the activity of NF-κB and possibly other signaling pathways.

## Introduction

Pirin is an iron-binding protein belonging to the functionally diverse cupin superfamily of proteins [1]. It was originally described as a nuclear protein, but subsequent studies showed that it could also be found in the cytoplasm [2, 3]. So far two functions of pirin have been proposed. The first is related to its putative enzymatic activity—the bacterial ortholog of pirin was found to be capable of oxidizing flavonoid quercetin in vitro [4]. Nevertheless, the biological significance of pirin’s quercetinase activity in mammalian cells remains uncertain. The second function of pirin is transcription coregulation—pirin was originally isolated as an interactor of the NFIX transcription factor [3] and was subsequently revealed also to form complexes with BCL3 and NF-κB1 (p50) [5], as well as RelA (p65) subunit of the NF-κB transcription factor [6]. Therefore, it may be involved in the regulation of the NF-κB-related transcription. It has been shown that pirin-BCL3 interaction is important for the regulation of *SNAI2* expression and that the inhibition of this interaction resulted in the decreased migration of melanoma cells [7].

The importance of the cellular functions of pirin is highlighted by the fact that changes in pirin expression were observed in several human cancers including acute myeloid leukemia [8], melanoma [2, 7], and colorectal carcinoma [9]. Several studies reported an upregulation of *PIR* expression by cigarette smoke in the airway epithelial cells [10, 11] and one of them linked pirin with apoptosis [12]. Regardless of the final cellular outcome of pirin activity, the following observations point to pirin as a positive rather than a negative regulator of transcription: (i) *SNAI2* expression was decreased after pirin inhibition [7]; (ii) NF-κB induction after TNFα treatment was significantly higher in the pirin-overexpressing HeLa cells compared to the control cells [13]; (iii) negative genetic interaction between *PIR* and histone deacetylase *HDAC2* was reported [14]; (iv) spectroscopic results showed that the ferric form of pirin facilitates binding of NF-κB proteins to target κB sequences in vitro [6].

If we think of pirin as a messenger modulating activity of NFIX, NF-κB and possibly other transcription factors, the question arises: who is sending the message? In other words: how is pirin activity and expression regulated? It is known that the human *PIR* gene is expressed at different levels in various organs (heart, brain, liver, kidney, lung, pancreas, placenta, and skeletal muscle); the highest expression is observed in the liver and heart, while the lowest in the brain and pancreas [3]. Hübner et al. [10] reported that in the small airway epithelium *PIR* expression correlates with NRF2 (NFE2L2) activity and suggested that *PIR* is one of NRF2-dependent genes. This hypothesis was supported by CHIP-seq data published by Chorley et al. [15]. Literature data also suggest that AP-1 [16] and NF-κB [8] transcription factors are potentially involved in the modulation of *PIR* expression. Since all these factors are activated in response to oxidative stress, their involvement in *PIR* expression is in line with our previous results showing increased *Pir* mRNA level in Sod1-deficient mice [17].

In this paper, we present the results of our analysis of the human *PIR* gene promoter. These data clearly indicate that the short region located downstream of the transcription start site (TSS), and containing the functional antioxidant response element (ARE; the binding site for the NRF2 transcription factor), is crucial for *PIR* expression in HeLa cells. Our experiments support the concept that *PIR* expression is highly dependent on NRF2 activity, and we hypothesize that pirin enables cross talk between NRF2 and other transcription factors.

## Materials and methods

### Cell culture and tBHQ treatment

Human cervical adenocarcinoma cells (HeLa) were grown in Quantum 101 medium (PAA). Asynchronous cell cultures in the exponential phase of growth were used in all experiments.* Tert*-Butylhydroquinone (tBHQ) (Sigma-Aldrich) was dissolved in dimethylsulphoxide to produce a 100 mM stock solution. The stock solution was diluted in Quantum 101 medium to give concentrations of 10 and 25 μM. Control cells were treated with dimethylsulphoxide diluted in Quantum 101 similar to tBHQ stock solution.

### Construction of the reporter plasmids with luciferase transcription under the control of *PIR* promoter sequences

Genomic DNA was isolated from the human cell line K562 (myelogenous leukemia) using a Genomic Mini kit (A&A Biotechnology) according to the manufacturer’s protocol. 1,729 bp DNA fragment was amplified by PCR using high Fidelity Phusion DNA polymerase (New England Biolabs) and the following primers: forward 5′-AGCCTTGAACTGCCTAAGTA-3′ (1,033 bp upstream of TSS of *PIR* gene), reverse 5′ ATCACCTACATCGAAGCAAC 3′ (696 downstream of TSS of *PIR* gene). The cycling conditions were as follows: initial denaturation at 98 °C for 1 min 15 s, followed by 33 cycles of denaturation at 98 °C for 8 s, annealing at 63 °C for 30 s and elongation at 72 °C for 51 s, followed by final elongation at 72 °C for 8 min. The PCR product was subsequently blunt-end ligated into pUC19 plasmid and sequenced. The resulting construct served as a template for the generation of the truncated *PIR* promoter sequences that were amplified using combinations of nine forward primers engineered to include *Xho*I site (P1F-P9F) and four reversed primers engineered to include *Hin*dIII site (P1R-P4R). The sequences of the primers are shown in Table 1. The amplified products were ligated into *Xho*I–*Hin*dIII double digested pGL4.10 plasmid (Promega).

**Table 1:** The sequences and genomic localizations of the primers used to generate the *PIR* promoter deletion constructs

Primer name	Primer sequence (5′–3′)	Distance from 5′ primer end to *PIR* TSS (bp)
P1F	atttctcgAGCCCACTACCTATTTTTGTATAGC	−885
P2F	aactcGAGAATGTACATGGCCTGC	−641
P3F	tttctcGAGACACCACTGTCTTTCC	−564
P4F	atactcgAGGCAGGAATAAAGACGTATGG	−324
P5F	atctcGAGGAACCAGAGGCACAG	−128
P6F	ttactcgAGATTTCCCACAAGACCG	+10
P7F	atctcGAGCACAGCAAGTGCC	+76
P8F	tactcgAGCTGGCCTGGGAG	+156
P9F	atactCGAGACCCGTAGACTCCCGC	+231
P1R	tttaagcTTCCCTCACCTAGTGGACC	+441
P2R	ttaaagcTTAAGAGAGTGTGGGTCCAGTAGC	+320
P4R	ataagcttCTAGAGGAGGCGGGAGGC	+263
P5R	attaagctTCCCAGGCCAGCTTGG	+168

The nongenomic overhangs are presented as small letters; the fragments corresponding to the genomic sequence are shown in capitals. The TSS localization according to the *PIR* mRNA sequence NM_001018109

### Cloning of the antioxidant response element cassettes into a minimal promoter luciferase reporter vector pGL4.23

Twenty-five base pairs long single stranded DNA oligonucleotides identical to the core AREs with their surrounding sequences, and their complementary sequences were synthesized commercially (DNA Sequencing and Oligonucleotide Synthesis Laboratory, Institute of Biochemistry and Biophysics, Warsaw, Poland). Equimolar mixtures of complementary DNA oligonucleotides in 10 mM Tris pH 8.0 were heated to 95 °C and chilled slowly to room temperature to enable the formation of ARE dsDNA cassettes, which were subsequently blunt-end ligated into EcoICRI digested pGL4.23 minimal promoter plasmid (Promega). The presence of the correct insert was verified by sequencing. Sequences of ARE cassettes are shown in Table 2.

**Table 2:** The sequences and localization of the ARE cassettes used in the study

ARE cassette sequence	Position relative to TSS (our designation)	Designation from Hübner et al. [10]
5′-CAGTCACA**GTGACTCAGCA**GAATCT-3′	−477 *NQO1*	–
3′-GTCAGTGT**CACTGAGTCGT**CTTAGA-5′		
5′-CGCGAAG**CGCTGAGTCAC**GGTGAGG-3′	+281 *PIR*	+33
3′-GCGCTTC**GCGACTCAGTG**CCACTCC-5′		
5′-CATGGCC**TGCAAAGTCAA**AGTATTT-3′	−625 *PIR*	–
3′-GTACCGG**ACGTTTCAGTT**TCATAAA-5′		
5′-CTGTATT**TGCTTTGTCAT**ATATCAA-3′	−2962 *PIR*	−3209
3′-GACATAA**ACGAAACAGTA**TATAGTT-5′		
5′-TTTGGAA**GTGATCTTGCA**GCTTGGA-3′	−3233 *PIR*	−3480
3′-AAACCTT**CACTAGAACGT**CGAACCT-5′		
5′-TATACTC**TGCATTGTCAT**CTTTACT-3′		
3′-ATATGAG**ACGTAACAGTA**GAAATGA-5′	−5219 *PIR*	−5466

Sequences matching the ARE consensus are given in bold

### Transfection

For transient plasmid DNA transfection Lipofectamine LTX reagent (Invitrogen) was used according to the manufacturer’s protocol. 24 h before the transfection the HeLa cells were seeded on 24-well cell culture plates at a density of 4 × 10^4^ cells per well in 0.5 ml of complete growth medium. The transfection was performed using 1 μl of Lipofectamine LTX, 250 ng of firefly luciferase reporter plasmid and, to normalize for transfection efficiency, 12.5 ng of pGL4.74 Renilla luciferase plasmid (Promega) per well. In the experiment involving NRF2 overexpression cells were transfected with pcDNA3-EGFP-C4-Nrf2 plasmid [18] (Addgene plasmid 21549) or pEGFP-N2 plasmid (Clontech) as a control.

In experiments involving siRNA transfection siPORT NeoFX Transfection Agent (Life Technologies) was used according to the manufacturer’s protocol. The final siRNA concentration was 5 nM. Silencer Select siRNA (Life Technologies) targeting NRF2 (siRNA ID: s9493) or scrambled siRNA (Silencer Select Negative Control No. 1 siRNA, catalog# 4390843) were used.

In experiments involving siRNA and plasmid DNA cotransfection cells were reverse transfected using siPORT NeoFX Transfection Agent (Life Technologies). Cells were trypsinized and diluted in growth medium. siPORT NeoFX Transfection Agent (1 μl per well) was diluted in Opti-MEM medium (Life Technologies) and incubated 10 min at room temperature. Plasmids (125 ng of firefly luciferase reporter plasmid and 6.25 ng of pGL4.74 Renilla luciferase plasmid per well) and Silencer Select siRNA (Life Technologies, 2.5 pmol per well, 5 nM final concentration) were diluted in Opti-MEM, mixed with diluted siPORT NeoFX Transfection Agent, incubated 10 min at room temperature and dispensed into a 24-well culture plate (50 μl per well). Subsequently, 450 μl of cell suspension (4 × 10^4^ cells) was added to each well and incubated under normal cell culture conditions.

### Dual-luciferase assay

24 h after the transfection, the cells were lysed, and the activities of the firefly and *Renilla* luciferases were measured using the Dual-Luciferase Reporter Assay System (Promega) on a TD-20/20 luminometer (Turner Biosystems) according to the manufacturers’ instructions.

### RNA isolation, reverse transcription, and real-time PCR

The total RNA was extracted from the cells using the RNeasy Plus Mini Kit (Qiagen) according to the manufacturer’s protocol. One microgram of total RNA was converted to cDNA in a 20 μl reaction volume using the High Capacity cDNA Reverse Transcription Kit (Life Technologies) following the manufacturer’s instructions. After reaction, cDNA was diluted to 100 μl with de-ionized, nuclease-free H_2_O. Real-time PCR was performed in a 20 μl reaction mixture containing 5 μl of diluted cDNA, 4 μl of de-ionized, nuclease-free H_2_O, 10 μl of TaqMan Gene Expression Master Mix (Life Technologies), and 1 μl of TaqMan Gene Expression Assay (Life Technologies). The following TaqMan assays were used: Hs00975961_g1 (*NRF2*), Hs01125825_m1 (*PIR* transcript variants 1 and 2, NM_003662.3 and NM_001018109.2 respectively), Hs01128656_m1 (only *PIR* transcript variant 1, NM_003662.3), Hs00168547_m1 (*NQO1*), and Hs01003267_m1 (*HPRT1*). All reactions were run in duplicate. PCR amplification was carried out using a 7500 Real-Time PCR System (Life Technologies) with an initial 10-min step at 95 °C followed by 40 cycles of 95 °C for 15 s and 60 °C for 1 min. Relative gene expression was calculated using the ΔΔCt method with *HPRT1* as a reference control.

### Chromatin immunoprecipitation (ChIP)

1 × 10^7^ HeLa cells were cross-linked by 1 % formaldehyde treatment for 10 min at room temperature. Crosslinking was stopped by adding glycine to a final concentration of 125 mM and incubating for 5 min at room temperature. The cells were washed twice with cold PBS, scraped and lysed in 1 ml of FA Lysis Buffer: 50 mM HEPES–KOH pH 7.5, 140 mM NaCl, 1 mM EDTA, 1 % Triton X-100, 0.1 % sodium deoxycholate, 0.1 % SDS and protease inhibitors (Complete, EDTA-free protease inhibitor cocktail tablets; Roche). Cross-linked chromatin was sonicated on ice for 10 min (15 s pulses, separated by 25 s rest) at 60 % amplitude using Vibra Cell VCX130 sonicator (Sonics) equipped with 2 mm microtip. Chromatin fragments of 200–1,000 bp were obtained. For each immunoprecipitation, 100 μl of sonicated chromatin was diluted 1:10 with RIPA buffer (50 mM Tris–HCl pH 8.0, 150 mM NaCl, 2 mM EDTA, 1 % NP-40, 0.5 % sodium deoxycholate, 0.1 % SDS, protease inhibitors). Twenty microliters of Protein A/G PLUS-Agarose beads (Santa Cruz Biotechnology, sc-2003) pretreated with BSA and low molecular DNA from salmon sperm, 5 μg of NRF2 rabbit polyclonal IgG (Santa Cruz Biotechnology, sc-722X), or normal rabbit IgG (Santa Cruz Biotechnology, sc-2027) were added and the sample was incubated overnight with the rotation at 4 °C. The next day, the beads were washed three times with a wash buffer (20 mM Tris–HCl pH 8.0, 150 mM NaCl, 2 mM EDTA, 1 % Triton X-100, 0.1 % SDS), once with a final wash buffer (20 mM Tris–HCl pH 8.0, 500 mM NaCl, 2 mM EDTA, 1 % Triton X-100, 0.1 % SDS) and once with a TE buffer. Subsequently, 100 μl of 10 % (wt/vol) Chelex 100 slurry (Bio-Rad) was added to the beads, followed by incubation at 100 °C for 15 min. The samples were then treated with Proteinase K (Qiagen) at 55 °C for 30 min and incubated again at 100 °C for 15 min. The samples were centrifuged and the supernatant was collected. The beads were resuspended in 120 μl of de-ionized, nuclease-free H_2_O, centrifuged again, and the supernatant was again collected and pooled with the supernatant collected previously. Real-time PCR was performed in a 20 μl reaction mixture containing 5 μl of the obtained supernatant, 3.8 μl of de-ionized, nuclease-free H_2_O, 10 μl of FastStart Universal SYBR Green Master (Rox) (Roche), and 0.6 μl of each 10 μM primer (final conc. 300 nM). All the primers used in the ChIP are listed in Table 3. All the reactions were run in triplicate. PCR amplification was carried out using 7500 Real-Time PCR System (Life Technologies) with an initial 10-min step at 95 °C followed by 40 cycles of 95 °C for 15 s and 60 °C (65 °C when CHIP281F and CHIP281R primers were used) for 1 min. The relative occupancy of the NRF2 at each *PIR* ARE was calculated as NRF2/IgG signal ratio and then normalized to the signal ratio observed for *NQO1* ARE.

**Table 3:** The sequences of primers used in the ChIP assay with the corresponding ARE

Primer name	Primer sequence (5′–3′)	ARE
NQO1AREF	CTTCCAAATCCGCAGTCACA	*NQO1*-477
NQO1ARER	AGCCTTGGCACGAAATGG	
CHIP281F	AAGCGCTGAGTCACGGTGAG	*PIR* +281
CHIP281R	AGCATTCCCTCACCTAGTGGAC	
CHIP625F	TGGCCTGCAAAGTCAAAGTATTT	*PIR* −625
CHIP625R	CATAGCTGCAGTTTCTATTCTCTAAACAC	
CHP2962F	TCCTTCTAGTTCTGATTCCCACTGT	*PIR* −2962
CHP2962R	AAACGGATTGATATATGACAAAGCAA	
CHP3233F	AAATAAATCACCAACTCATACTCTGGAA	*PIR* −3233
CHP3233R	TCCAACTCTAGCACCTTGTACACAGT	
CHP5219F	ACTCTGCATTGTCATCTTTACTCAGTTAG	*PIR −*5219
CHP5219R	CCCATGCCATGTCCCTTTAG	

### Western blot

24 h after siRNA transfection cytoplasmic and nuclear protein extracts were made using EpiSeeker Nuclear Extraction Kit (Abcam). Total protein in each fraction was determined by the modified Bradford assay [19]. 25 μg of total protein from each sample was separated on 12 % SDS–polyacrylamide gel and then electroblotted onto Immun-Blot PVDF Membrane (Bio-Rad). The membranes were stained with Ponceau S (Sigma-Aldrich) to verify equal amounts of sample loading and then incubated for 1 h at 4 °C in TBS-T with 5 % nonfat dry milk. The membranes were probed overnight at 4 °C with a specific primary antibody and then with a horseradish peroxidase conjugated secondary antibody The bound antibodies were detected by chemiluminescence using WesternBright ECL Chemiluminescence HRP Substrate (Advansta) and CL-XPosure Film (Thermo Scientific). After chemiluminescence detection antibodies were stripped using Restore Western Blot Stripping Buffer (Thermo Scientific) and the membranes were reprobed with different antibodies. The following primary antibodies were used: anti-Pirin ab21202 1:600 (Abcam), anti-Lamin B (C-20) sc-6216 1:500 (Santa Cruz Biotechnology), anti-α Tubulin (B-5-1-2) sc-23948 1:2000 (Santa Cruz Biotechnology).

### Statistical analyses

The results are presented as medians together with the maximum and minimum values. The statistical comparisons were performed by the Kruskal–Wallis one way ANOVA or Mann–Whitney* U* test. A value of *p* < 0.05 was considered statistically significant. All statistical analyses were performed using Statistica 7 software (StatSoft).

## Results

### *In silico* analysis of the region upstream and downstream of human *PIR* gene TSS

To search for the putative transcription regulatory sequences in the *PIR* gene promoter we analyzed the sequence ranging from 900 bp upstream to 500 bp downstream of the putative *PIR* TSS (5′ end of *PIR* transcripts NM_003662.3 and NM_001018109.2). In order to predict the type of core promoter (sharp or broad) we searched for the presence of canonical core promoter elements using the JASPAR POLII database—the collection of models describing patterns found in RNA Polymerase II promoters [20] (http://jaspar.genereg.net/). We have found the potential initiator element (Inr) that consists of the sequence ACAGTTAA (the underlined position corresponds to +1). This potential Inr doesn’t entirely match the classical Inr consensus sequence (YYANWYY), but it includes the pyrimidine–purine (PyPu) dinucleotide consensus in positions −1, +1 which shows strong conservation over eukaryotic core promoters. We have also identified the potential downstream promoter element (DPE) that consists of the sequence AGACC starting at position +23 but its functional relevance is uncertain, since DPE is typically located from +28 to +32 [21]. We did not find any potential TATA box near the TSS.

CpG islands are genomic regions often associated with the transcription initiation site in which CG dinucleotides are overrepresented. A genome-wide analysis has shown that 72 % of human promoters are associated with CpG islands [21]. Using the CpGplot program [22] (http://www.ebi.ac.uk/Tools/emboss/cpgplot/) we looked for the regions rich in the CpG pattern in the sequence surrounding the *PIR* TSS. A putative CpG island has been found in the region between positions +120/+386 with the following features: size = 267 bp, sum C + G = 188 % CG = 70.41, observed/expected ratio = 0.76.

**Fig. 1:**
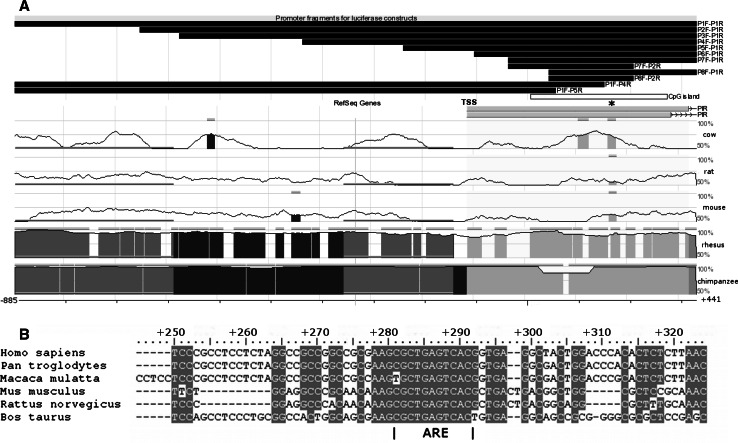
**a** The conservation of the *PIR* promoter sequence in the genomes of the chimpanzee, rhesus monkey, mouse, rat, and cow in relation to the human. The following features are marked: the TSS; the most conserved region containing putative ARE (*asterisk*); the predicted CpG island (*white bar*); the promoter fragments used in the luciferase constructs (*black bars*). **b** The enlargement of the alignment of DNA region marked on (**a**) with *asterisk*. The putative ARE, located between positions +281/+291 in the human gene, is shown

Then, we used the ECR browser [23] (http://ecrbrowser.dcode.org) to analyze the conservation of the sequence upstream and downstream *PIR* TSS in the following species: human (*Homo sapiens*), chimpanzee (*Pan troglodytes*), rhesus monkey (*Macaca mulatta*), mouse (*Mus musculus*), rat (*Rattus norvegicus*), and cow (*Bos taurus*). We have identified a highly conserved 15-bp-long element which is almost identical among all the species tested. It is the most conserved sequence in the *PIR* promoter region; in the human it starts 278 bp downstream of the TSS (Fig. 1a). Using the Jaspar Core Vertebrata database [20] (http://jaspar.genereg.net/), we checked whether the sequence included any known transcription factor binding sites, and we concluded that it may contain an ARE—a binding site for the NRF2 transcription factor, located between positions +281/+291 (Fig. 1b).

### The conserved region containing putative ARE is the crucial part of *PIR* promoter

**Fig. 2:**
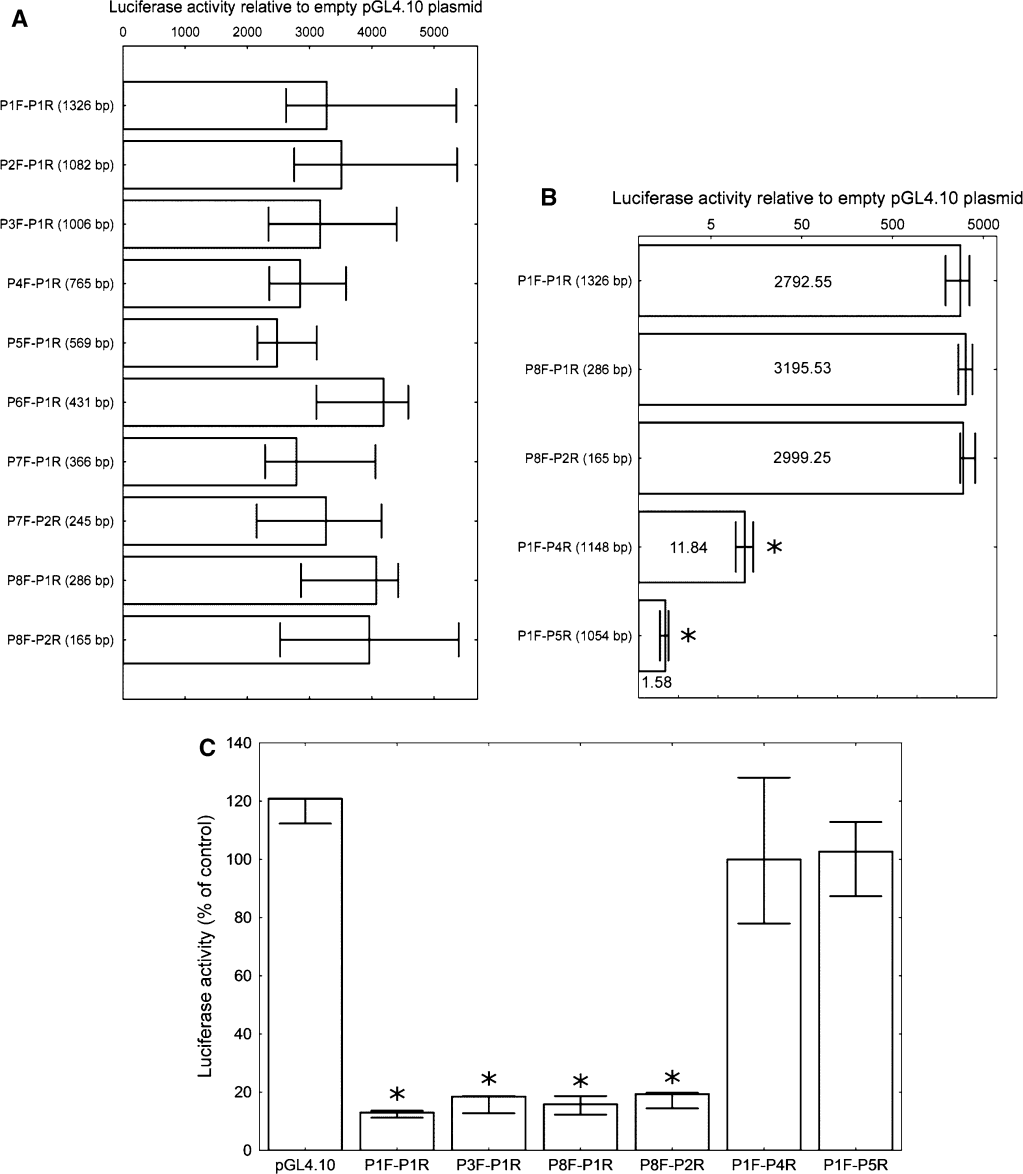
The luciferase activity in HeLa cells transfected with pGL4.10 plasmid bearing different *PIR* promoter fragments. The firefly luciferase activity was normalized to *Renilla* luciferase activity and is shown as the fold changes in activity compared to the empty vector. The *columns* represent the median values and the *bars* represent the minimum and maximum values from four independent experiments. **a** Ten constructs containing the *PIR* promoter fragments of different lengths (given in parentheses), each of which contains the putative ARE from position +281. The constructs with length ≥569 bp contain both potential Inr and DPE elements, P6F-P1R contains only DPE while the rest contain neither Inr nor DPE. The differences are not statistically significant in Kruskal–Wallis one way ANOVA. **b** Three constructs containing ARE +281 (P1F-P1R, P8F-P1R, and P8F-P2R) and two constructs lacking ARE +281 (P1F-P4R, P1F-P5R). The values are presented on a logarithmic scale. The exact median value for each construct is depicted on the respective column. The *asterisks* denote a statistically significant difference in the Mann–Whitney* U* test between the respective construct and all the other constructs. **c** The luciferase expression in HeLa cells transfected with the NRF2-targeted siRNA relative to the control (scrambled siRNA transfected cells). Four plasmids containing the putative ARE +281 (P1F-P1R, P3F-P1R, P8F-P1R, and P8F-P2R), two plasmids lacking the ARE +281 (P1F-P4R, P1F-P5R) and the empty pGL4.10 plasmid were used. The *asterisks* denote a statistically significant difference in the Mann–Whitney* U* test between the luciferase activity in the cells transfected with the NRF2-targeted siRNA and the cells transfected with scrambled siRNA

To find whether the analyzed promoter region and identified putative regulatory elements are functional in vivo, ten plasmids were constructed containing different fragments of the analyzed region cloned upstream of the firefly luciferase reporter gene *luc2* in a pGL4.10 plasmid (Promega) which lacks any known eukaryotic promoter elements. The longest *PIR* promoter fragment (named P1F-P1R from the names of the primers used for its amplification) was 1,326-bp-long and ranged from position −885 to +441. The other constructs contained various 5′ and 3′ deletion variants of the longest fragment as depicted in Fig. 1a. The shortest fragment (P8F-P2R) contained the central part of the predicted CpG island together with a putative ARE. The activities of the promoter fragments were measured as the ability to drive the expression of the *luc2* gene after transfection into the HeLa cells. To our surprise, all the fragments increased the luciferase activity to a similar extent, i.e., 3,000–4,000-fold compared to the empty pGL4.10 vector (Fig. 2a). There was no statistically significant difference between the activities of different promoter fragments. We concluded that the sequence responsible for the entire observed activity of the *PIR* promoter is located within the shortest fragment used in this experiment (165-bp long; from +156 to +320). To verify this conclusion, we constructed two additional plasmids. Plasmid P1F-P4R lacked the putative ARE element, but still included 5′ part of the P8F-P2R fragment, whereas plasmid P1F-P5R did not include any sequence from P8F-P2R (Fig. 1a). As expected, the absence of ARE resulted in a dramatic decrease in luciferase activity (about 250 times less), but still the P1F-P4R fragment was able to increase the luciferase activity about ten times relative to the empty plasmid. Nevertheless, it seems that the remaining activity was connected with the remaining part of P8F-P2R, which was present in P1F-P4R, since the P1F-P5R fragment caused only a slight increase (1.58-fold median) in luciferase activity relative to the empty plasmid (Fig. 2b).

If the hypothesis that NRF2 drives *PIR* expression via the highly conserved ARE in position +281 was correct, the depletion of NRF2 should have resulted in decreased *luc2* expression from constructs containing the ARE, while the expression from constructs without it should remain unchanged. We tested this hypothesis by comparing the luciferase expression from the ARE ± constructs in the HeLa cells transfected with either NRF2-targeted siRNA or scrambled siRNA as a control. As expected, the luciferase expression from the ARE containing constructs was considerably (more than 80 %) lower in cells transfected with anti-NRF2 siRNA compared to the control cells. In agreement, the expression from constructs without the ARE was unaffected by anti-NRF2 siRNA treatment (Fig. 2c).

### NRF2 silencing reduce pirin expression at mRNA and protein level

**Fig. 3:**
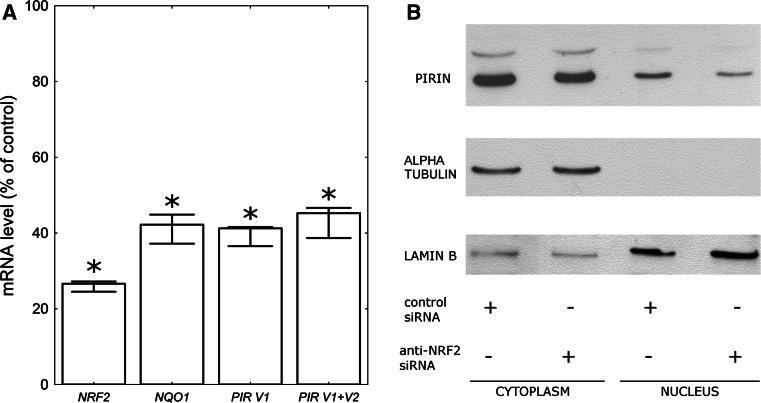
**a** *NRF2*, *NQO1*, and *PIR* mRNA level in the HeLa cells transfected with NRF2-targeted siRNA as a percent of the respective mRNA level in the control cells (scrambled siRNA transfected). The *columns* represent median values, while the *bars* represent minimum and maximum values from three independent experiments. The *asterisks* denote a statistically significant difference in the Mann–Whitney* U* test between the mRNA levels in cells transfected with NRF2-targeted siRNA and the control cells. **b** Western blot analysis of Pirin protein level in the cytoplasmic and nuclear extracts from the HeLa cells transfected with NRF2-targeted siRNA. Alpha Tubulin is shown as a marker of cytoplasmic fraction and Lamin B as a marker of nuclear fraction

To further confirm the dependence of *PIR* expression on NRF2 activity we used real-time PCR to compare the *PIR* mRNA level in the HeLa cells transfected with anti-NRF2 or scrambled, nontargeting siRNA. In this experiment we used 4 TaqMan assays: the first for the detection of both *PIR* transcripts; the second for the detection of only the longer *PIR* transcript (variant 1); the third for the detection of transcripts of *NQO1*—a gene well known to be NRF2 dependent, used here as a positive control; and the fourth for the detection of *NRF2* transcript. The results are shown in Fig. 3a. A 70 % decrease in *NRF2* expression resulted in about a 60 % down-regulation of both *NQO1* and *PIR*. Both TaqMan assays for *PIR* gave similar results. This indicates that both *PIR* transcripts were affected to a similar extent. By comparing the results obtained for both *PIR* TaqMan assays we have calculated that in HeLa cells the longer transcript constitutes ~40 % of all *PIR* transcripts. Wendler et al. [3] reported that about 15 % of *PIR* cDNAs isolated from HeLa cells during their study were longer transcripts containing a short 34-bp extra element within the 5′-UTR and assigned as transcript variant 1. Our result suggests that the longer *PIR* transcript in HeLa cells may be much more abundant than could be expected based on the previous report.

Western blot analysis showed that also the pirin protein level decreased both in the cytoplasm and nucleus of cells transfected with anti-NRF2 siRNA (Fig. 3b).

### tBHQ treatment and NRF2 overexpression have little effect on *PIR* expression in HeLa cells

**Fig. 4:**
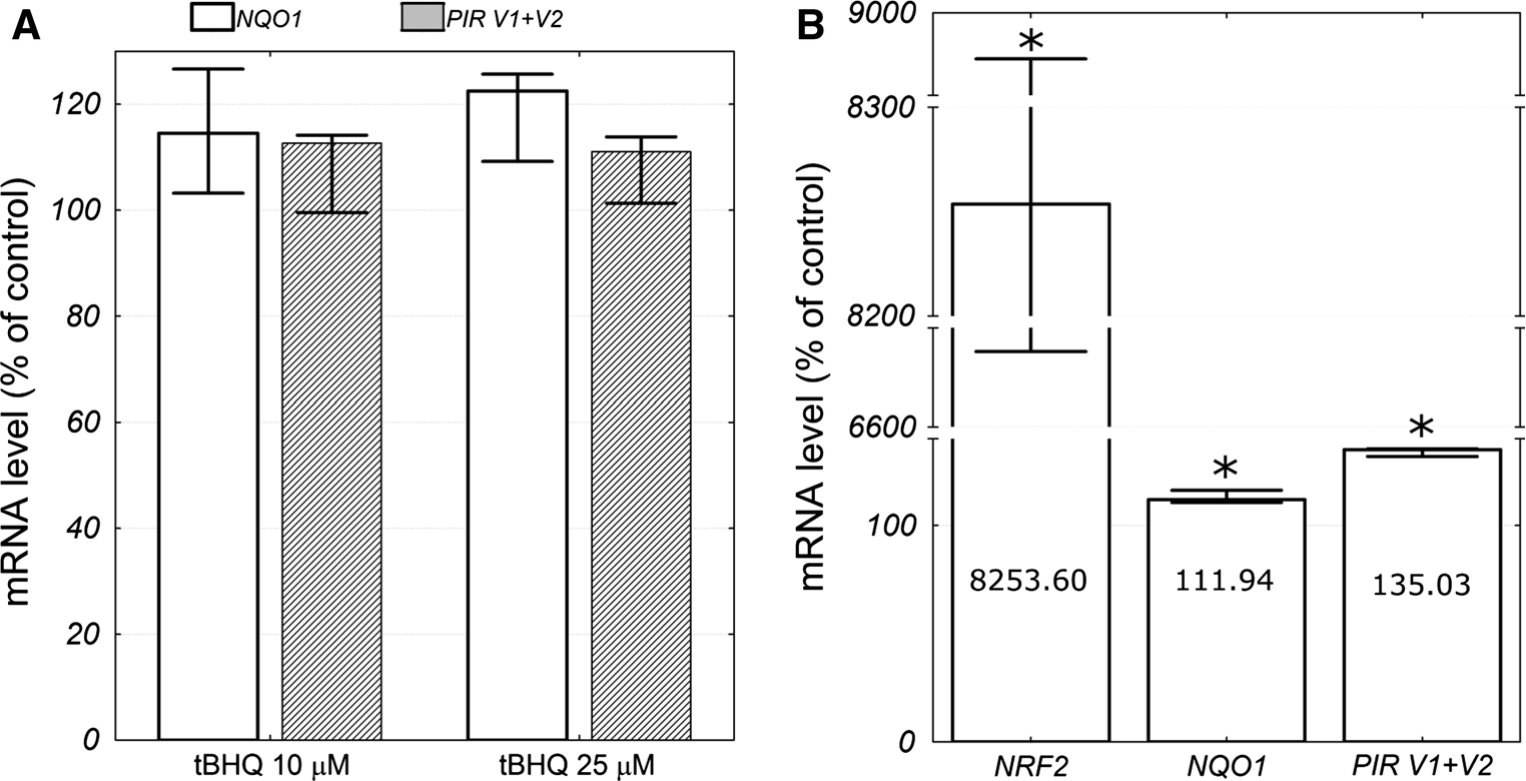
**a** *NQO1* and *PIR* mRNA level in the HeLa cells 5 h after treatment with 10 or 25 μM tBHQ as a percent of the respective mRNA level in the control cells. The *columns* represent median values, while the *bars* represent minimum and maximum values from three independent experiments. Differences were not statistically significant in Kruskal–Wallis one way ANOVA. **b** *NRF2*, *NQO1*, and *PIR* mRNA level in the HeLa cells transfected with pcDNA3-EGFP-C4-Nrf2 plasmid as a percent of the respective mRNA level in the control cells (pEGFP-N2 transfected). The *columns* represent median values, while the *bars* represent minimum and maximum values from three independent experiments. The exact median value for each gene is depicted on the respective column. The *asterisks* denote a statistically significant difference in the Mann–Whitney* U* test between the mRNA levels in cells transfected with pcDNA3-EGFP-C4-Nrf2 plasmid the control cells

In the absence of cellular stress NRF2 is sequestered in the cytoplasm through interaction with KEAP1 and directed to proteosomal degradation. To check if NRF2 activation affects *PIR* expression we treated HeLa cells with tBHQ—a known NRF2 activator [24]. Real-time PCR analysis showed that neither *PIR* nor *NQO1* expression was significantly affected by tBHQ treatment (Fig. 4a). To further check if ectopic NRF2 overexpression will affect *PIR* expression we transfected HeLa cells with pcDNA3-EGFP-C4-Nrf2 plasmid expressing EGFP-NRF2 fusion protein. EGFP-NRF2 overexpression caused slight, but statistically significant increase in *PIR* and *NQO1* mRNA level relative to control cells expressing EGFP protein (Fig. 4b).

### Only ARE in position +281 in *PIR* promoter is a functional binding site for NRF2 in HeLa cells

Hübner et al. [10] observed that *PIR* expression in the human small airway epithelium is correlated with NRF2 activity and identified four potential ARE elements in the *PIR* promoter, two of which seemed to be functional based on the electrophoretic mobility shift assay. Because of the different putatives TSS used by Hübner et al. to describe the localization of potential AREs, the designations used by those authors are different from the ones used in our study. The sequences of putative AREs from the *PIR* promoter together with the designations used by Hübner et al. and by us are summarized in Table 2. In addition to the ARE identified by Hubner et al. we have identified previously undescribed ARE in position −625. The reason why Hubner et al. did not identify this element is likely due to the different software used for the analysis. Hubner et al. used Genamics Expression 1.1 Pattern Finder Tool Software and we used the Jaspar Core Vertebrata database. Different software may use slightly different consensus sequences or matrix models for transcription factor binding sites and this is probably the reason for the discrepancies. The ARE in position +33 from the study of Hübner et al. corresponds to the highly conserved ARE which we identified in position +281, as described above. “Interestingly, in in vitro electrophoretic mobility shift assay” AREs +281 and −3233 did not prove to be functional, whereas AREs −2962 and −5219 were functional [10].

**Fig. 5:**
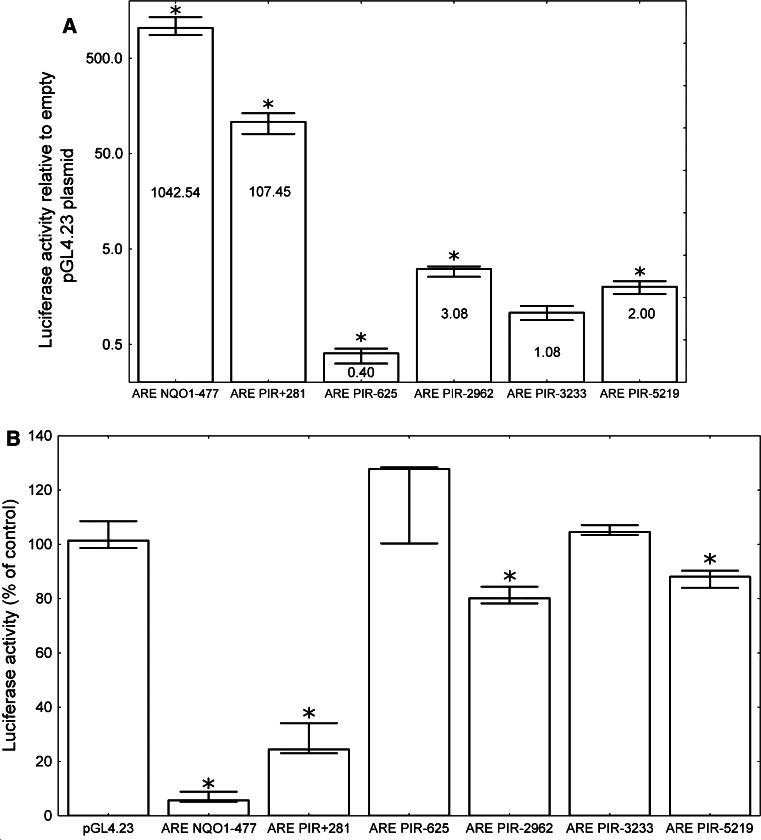
The activities of putative AREs from *PIR* in comparison to the activity of *NQO1* ARE. The *columns* represent median values and the *bars* represent the minimum and maximum values from four independent experiments. **a** The HeLa cells were transfected with pGL4.23 plasmids containing various ARE cassettes. The firefly luciferase activity was normalized to the *Renilla* luciferase activity and is shown as a fold change in the activity relative to the empty vector. An *asterisk* denotes a statistically significant difference in the Mann–Whitney* U* test compared to the empty vector. Differences between plasmids are also statistically significant. **b** The HeLa cells were transfected with pGL4.23 plasmids containing various ARE cassettes together with NRF2-targeted or scrambled siRNA. The firefly luciferase activity was normalized to *Renilla* luciferase activity and is shown as a percent of the control (scrambled siRNA transfected cells). An *asterisk* denotes a statistically significant difference in the Mann–Whitney* U* test between the luciferase activity in the cells transfected with NRF2-targeted siRNA and control cells

Our results strongly suggest that ARE +281 is functional; therefore, we decided to analyze and compare the activities of the putative AREs from the *PIR* promoter identified by Hübner et al. plus the additional ARE which we identified in position −625 that was not analyzed by Hübner et al. To this end, we cloned 25 bp DNA cassettes containing each potential ARE upstream of a minimal promoter and the firefly luciferase reporter gene *luc2* in a pGL4.23 plasmid (Promega). We also made an analogous construct with a prototypical ARE from position −477 in the *NQO1* gene and used it as a positive control in the following experiments. Each construct was transfected into the HeLa cells, and the luciferase activity was analyzed 24 h after transfection. As expected, the highest luciferase activity was observed in the cells transfected with the construct containing *NQO1* ARE (~1,000-fold increase relative to the empty plasmid). The *PIR* +281 ARE was 10 times less active than the one from *NQO1,* but it still induced an ~100-fold increase in luciferase activity relative to the empty plasmid and proved to be the most active among the putative ARE elements found in the *PIR* promoter. The AREs from positions −2962 and −5219 increased luciferase activity only slightly (three and twofold, respectively), whereas ARE −3233 did not affect the *luc2* expression at all, and ARE −625 even caused a slight decrease in luciferase activity (Fig. 5a). To confirm that the observed AREs’ activities are dependent on NRF2, we repeated the above experiment in cells transfected with either NRF2-targeted or scrambled siRNA. As expected, *NQO1* ARE proved to be the most sensitive to NRF2 depletion, since its activity in the anti-NRF2 siRNA treated cells was <10 % of control. The luciferase activity from the *PIR* +281 ARE decreased by ~70 % after NRF2 silencing. Activities of AREs −2962 and −5219 were also decreased, but only by about 20 and 10 %, respectively. In line with the previous experiment, the putative AREs from positions −625 and −3233 did not respond to NRF2 silencing (Fig. 5b).

**Fig. 6:**
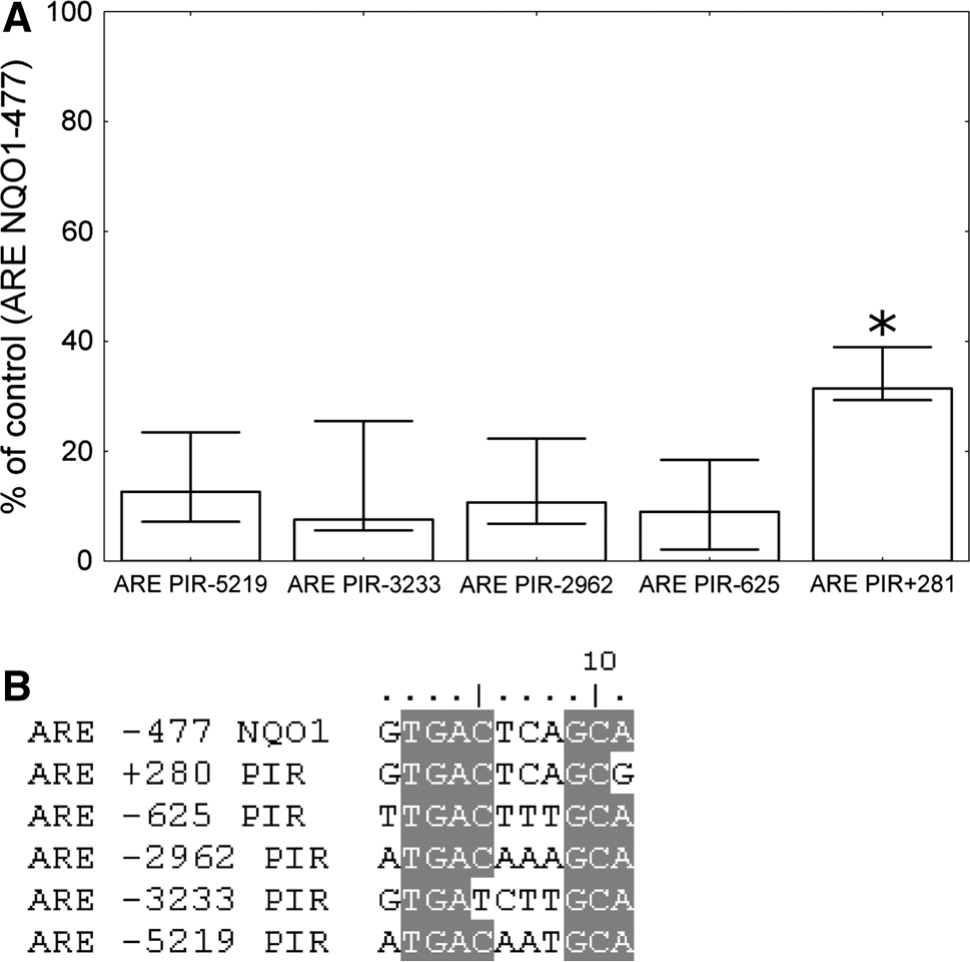
**a** ChIP analysis demonstrating the NRF2 binding to the *PIR* AREs. The assay was performed on the HeLa cells using the anti- NRF2 antibody and the normal rabbit IgG. Results are presented as NRF2/IgG ratio normalized to *NQO1* ARE. The *asterisk* denotes a statistically significant difference in the Mann–Whitney* U* test between the ARE +281 and the other AREs. The *columns* represent the median values, and the *bars* represent the minimum and maximum values from the four independent experiments. **b** The alignment of the five potential AREs from *PIR* and the ARE from *NQO1*

To validate in vivo NRF2 binding to the potential AREs in the *PIR* promoter in HeLa cells, we performed chromatin immunoprecipitation (ChIP) experiments with the anti-NRF2 antibody followed by real-time PCR with primers designed to amplify DNA fragments containing the predicted AREs and *NQO1* −477 ARE as a positive control (Table 3). The result of this experiment is shown in Fig. 6a. Taking into account that in the previous experiments the AREs −625 and −3233 proved not to be functional, in the ChIP analysis their signal is considered here as a background, the effect of unspecific binding, equal to ~10 % of the signal generated by primers specific to the *NQO1* ARE. The signals generated by primers specific to the AREs −5219 and −2962 were not significantly higher than the background defined above, whereas the primers specific to the ARE +281 generated a signal that was substantially higher than the background and equal to 30 % of the *NQO1* ARE’s signal.

Together, these experiments confirm that the ARE in position +281 in the *PIR* promoter, although less active than the “classical” ARE from *NQO1*, is fully functional, and the NRF2 transcription factor regulates the *PIR* expression in HeLa cells through this element.

## Discussion

Core promoters can be roughly divided into two classes: those having a single, sharply defined TSS, and those that have a very broad range of potential TSSs over a 50–100 bp region. Several common DNA sequence elements and patterns including TATA box, Inr, DPE, TFIIB recognition element (BRE), and CpG islands are associated with core promoters. The “sharp” promoters often contain a TATA box, while the “dispersed” (broad TSS distribution) core promoters usually consist of CpG islands. The first type of promoters is primarily used for tissue-specific expression, whereas the second is generally associated with ubiquitously expressed genes. Most of the genes in the higher eukaryotes are under the control of the dispersed core promoters [21, 25]. Although we have identified potential Inr and DPE elements near *PIR* TSS, our experiments did not prove their functionality, since all constructs showed similar luciferase expression regardless of the fact that only a part of them included the potential Inr and DPE elements (Fig. 1a). The region which we identified as crucial for *PIR* expression in HeLa cells lies downstream from the TSS, within the CpG island. The most conserved fragment of this region consists of the ARE (consensus sequence 5′-RTGAYNNNGC-3′)—a binding site for the NRF2 transcription factor.

The nuclear factor erythroid derived 2 like 2 (NRF2, NFE2L2) transcription factor is a Cap’n’Collar basic-region leucine zipper transcription factor that controls the cellular responsiveness to oxidants and electrophiles by inducing the expression of antioxidant and detoxification genes. The proteins coded by the NRF2 target genes have a variety of functions including direct oxidant inactivation, glutathione synthesis, NADPH regeneration, toxin export, the repair or removal of damaged proteins, and the inhibition of inflammation. NRF2 also regulates the expression of growth factors and their receptors, as well as various transcription factors [26]. The regulation of the expression of transcription factors is one form of the interaction between NRF2 and other transcription factors and signaling pathways. Other forms of such interaction include posttranslational modifications, competing for binding sites or transcription coactivators. A comprehensive review of the observations on the molecular interactions between NRF2 and the arylhydrocarbon receptor (AhR), NF-κB, p53, and Notch1 signaling pathways can be found in the paper of Wakabayashi et al. [27], while the hypotheses about the NRF2/KEAP1 relation to autophagy and the apoptosis pathways have been presented in our previous paper [28].

Pirin is a transcriptional coactivator whose influence on NF-κB dependent transcription via interaction with BCL3 was shown in the case of *SNAI2* expression in melanoma cells [7]. This observation is in line with our previous results showing a higher induction of NF-κB dependent luciferase expression after TNFα treatment in pirin-overexpressing cells than in control cells [13]. Taking into consideration the findings mentioned above and the results presented in this paper, proving that *PIR* expression is regulated by NRF2, one can hypothesize that pirin may act as a mediator of cross-talk between NRF2 and NF-κB (possibly also NFIX and other transcription factors and signaling pathways), and that NRF2 can influence the expression of NF-κB dependent genes by regulating *PIR* expression. Various mechanisms of NRF2—NF-κB cross-talk have been described to date. Many examples exist in which activation and repression occur between members of the two pathways. Several cancer chemopreventive agents trigger NRF2 signaling with a concomitant repression of NF-κB and its target genes. For example, epigallocatechin-3-gallate induces NRF2 and reduces the levels of NF-κB, TNFα, and IL-1β in the lungs of bleomycin-treated rats [29], while chalcone has been shown to induce NRF2 and inhibit NF-κB activation in endothelial cells [30]. A very interesting mechanism of NF-κB-NRF2 interaction was recently described: in endothelial cells upon TNFα treatment NF-κB activated microRNA miR-155 expression which led to the inhibition of BACH1 (repressor of the ARE-dependent HMOX1 transcription) translation and resulted in the NRF2-dependent activation of HMOX1 expression [31]. It was also reported that the p65 (RELA) subunit of NF-κB interacts with KEAP1; this results in decreased NRF2 binding to its cognate DNA sequences and enhanced NRF2 ubiquitination [32]. All such cross-talk between signaling pathways is crucial for the proper fine-tuning of the cellular response to stress conditions and its malfunction may result in abnormal cell growth and initiate or contribute to the process of carcinogenesis.

Liu et al. [6] proposed recently that pirin may act as a redox sensor for the NF-κB transcription factor. According to their model, pirin serves as a reversible functional switch that depends on the oxidation state of its iron cofactor and modulates NF-κB activity in response to the changes in redox level of the cell nucleus. From the previous work and our present study the interesting picture emerges in which pirin is regulated by the redox state of the cell at two levels. First is the regulation of *PIR* expression by the NRF2 transcription factor; and second is the modification of pirin’s activity by changes in the oxidation state of its iron cofactor.

Aside from the highly conserved ARE in position +281, four other potential AREs are present in the *PIR* gene. Using, the luciferase reporter system, and the ChIP assay we tested the activities of all potential AREs and compared them to the activity of a well-documented ARE from the *NQO1* promoter. We concluded that in the unstimulated HeLa cells NRF2 binds to and drives expression from ARE +281 only. This result is in contradiction to the in vitro electrophoretic mobility shift assay, performed by Hübner et al. [10], in which the AREs +281 and −3233 were not proved to be functional, whereas the AREs −2962 and −5219 were attributed as functional. This discrepancy may be due to the different methods used to analyze ARE functionality (EMSA in Hubner et al. and luciferase assay in our study) or different cellular contexts (Hubner et al. used nuclear extract from small airway epithelium in their experiments and we performed ours in HeLa cells). Furthermore, we cannot exclude the possibility that NRF2 is able to activate transcription through the AREs −5219, −3233, −2962, and −625 under specific conditions. Nevertheless, our results undeniably showed that the ARE in position +281 in the *PIR* gene is functional.

Sequences of all the AREs studied in this paper are compared in Fig. 6b. Based on our experiments, we cannot definitively explain the observed difference in activity between the ARE +281 and the ARE *NQO1* (approximately tenfold in the luciferase reporter assay as shown in Fig. 5a and threefold in the ChIP as shown in Fig. 6a). This difference may be due to (1) the difference in the last nucleotide, (2) the fact that the *NQO1* ARE is located on the sense strand of the *NQO1* gene, while the ARE +281 is located on the antisense strand of the *PIR* gene, (3) the differences in the sequences surrounding both the ARE elements. Unlike other AREs analyzed in this study, AREs *NQO1* and *PIR* +281 include AP-1 binding site with sequence TGACTCA. The regulation of *NQO1* by the AP-1 transcription factor through this site was confirmed and described [33, 34]. It is highly probable that AP-1 is also involved in the regulation of *PIR* expression, since pirin overexpression in *c*-*JUN* (subunit of AP-1 transcription factor)-transformed fibroblasts has been reported [16].

Our experiments with tBHQ treatment and NRF2 overexpression (Fig. 4) suggest that NRF2 is responsible mainly for basal and not inducible *PIR* expression in HeLa cells. NRF2 activation is a sophisticated process which can have different cellular outcomes depending on the nature of the activator and the cellular context. For example, in the study performed by Chorley et al. [15] NRF2 activator sulforaphane induced *PIR* expression in the BEAS-2B cells, but not in the A549 cells. In addition, different sets of genes can be activated by NRF2 in response to different stimuli.

Taken together, in this work we have proved that the basal *PIR* expression in HeLa cells is largely dependent on the NRF2 transcription factor which acts through a highly conserved ARE located 281 bp downstream of the TSS. We hypothesized that the regulation of the *PIR* expression may constitute a mechanism by which NRF2 is able to modulate the activity of NF-κB and possibly other transcription factors. Further experiments are necessary to test this hypothesis.

## References

[1] Pang H, Bartlam M, Zeng Q, Miyatake H, Hisano T, Miki K, Wong LL, Gao GF, Rao Z (2004). Crystal structure of human pirin: an iron-binding nuclear protein and transcription cofactor. J Biol Chem.

[2] Licciulli S, Luise C, Zanardi A, Giorgetti L, Viale G, Lanfrancone L, Carbone R, Alcalay M (2010). Pirin delocalization in melanoma progression identified by high content immuno-detection based approaches. BMC Cell Biol.

[3] Wendler WM, Kremmer E, Forster R, Winnacker EL (1997). Identification of pirin, a novel highly conserved nuclear protein. J Biol Chem.

[4] Adams M, Jia Z (2005). Structural and biochemical analysis reveal pirins to possess quercetinase activity. J Biol Chem.

[5] Dechend R, Hirano F, Lehmann K, Heissmeyer V, Ansieau S, Wulczyn FG, Scheidereit C, Leutz A (1999). The Bcl-3 oncoprotein acts as a bridging factor between NF-kappaB/Rel and nuclear co-regulators. Oncogene.

[6] Liu F, Rehmani I, Esaki S, Fu R, Chen L, de Serrano V, Liu A (2013). Pirin is an iron-dependent redox regulator of NF-kappaB. Proc Natl Acad Sci USA.

[7] Miyazaki I, Simizu S, Okumura H, Takagi S, Osada H (2010). A small-molecule inhibitor shows that pirin regulates migration of melanoma cells. Nat Chem Biol.

[8] Licciulli S, Cambiaghi V, Scafetta G, Gruszka AM, Alcalay M (2010). Pirin downregulation is a feature of AML and leads to impairment of terminal myeloid differentiation. Leukemia.

[9] Yoshikawa R, Yanagi H, Hashimoto-Tamaoki T, Morinaga T, Nakano Y, Noda M, Fujiwara Y, Okamura H, Yamamura T (2004). Gene expression in response to anti-tumour intervention by polysaccharide-K (PSK) in colorectal carcinoma cells. Oncol Rep.

[10] Hubner RH, Schwartz JD, De BP, Ferris B, Omberg L, Mezey JG, Hackett NR, Crystal RG (2009). Coordinate control of expression of Nrf2-modulated genes in the human small airway epithelium is highly responsive to cigarette smoking. Mol Med.

[11] Spira A, Beane J, Shah V, Liu G, Schembri F, Yang X, Palma J, Brody JS (2004). Effects of cigarette smoke on the human airway epithelial cell transcriptome. Proc Natl Acad Sci USA.

[12] Gelbman BD, Heguy A, O’Connor TP, Zabner J, Crystal RG (2007). Upregulation of pirin expression by chronic cigarette smoking is associated with bronchial epithelial cell apoptosis. Respir Res.

[13] Brzóska K, Kruszewski M (2009). Impact of pirin protein expression level on NF-kappaB signaling pathway activation. AIP Conf Proc.

[14] Lin YY, Kiihl S, Suhail Y, Liu SY, Chou YH, Kuang Z, Lu JY, Khor CN, Lin CL, Bader JS, Irizarry R, Boeke JD (2012). Functional dissection of lysine deacetylases reveals that HDAC1 and p300 regulate AMPK. Nature.

[15] Chorley BN, Campbell MR, Wang X, Karaca M, Sambandan D, Bangura F, Xue P, Pi J, Kleeberger SR, Bell DA (2012). Identification of novel NRF2-regulated genes by ChIP-Seq: influence on retinoid X receptor alpha. Nucleic Acids Res.

[16] Bergman AC, Alaiya AA, Wendler W, Binetruy B, Shoshan M, Sakaguchi K, Bergman T, Kronenwett U, Auer G, Appella E, Jornvall H, Linder S (1999). Protein kinase-dependent overexpression of the nuclear protein pirin in c-JUN and RAS transformed fibroblasts. Cell Mol Life Sci.

[17] Brzoska K, Stepkowski TM, Kruszewski M (2011). Putative proto-oncogene Pir expression is significantly up-regulated in the spleen and kidney of cytosolic superoxide dismutase-deficient mice. Redox Rep.

[18] Furukawa M, Xiong Y (2005). BTB protein Keap1 targets antioxidant transcription factor Nrf2 for ubiquitination by the Cullin 3-Roc1 ligase. Mol Cell Biol.

[19] Zor T, Selinger Z (1996). Linearization of the Bradford protein assay increases its sensitivity: theoretical and experimental studies. Anal Biochem.

[20] Bryne JC, Valen E, Tang MH, Marstrand T, Winther O, Krogh A, Lenhard B, Sandelin A (2008). JASPAR, the open access database of transcription factor-binding profiles: new content and tools in the 2008 update. Nucleic Acids Res.

[21] Sandelin A, Carninci P, Lenhard B, Ponjavic J, Hayashizaki Y, Hume DA (2007). Mammalian RNA polymerase II core promoters: insights from genome-wide studies. Nat Rev Genet.

[22] Larsen F, Gundersen G, Lopez R, Prydz H (1992). CpG islands as gene markers in the human genome. Genomics.

[23] Ovcharenko I, Nobrega MA, Loots GG, Stubbs L (2004). ECR Browser: a tool for visualizing and accessing data from comparisons of multiple vertebrate genomes. Nucleic Acids Res.

[24] Imhoff BR, Hansen JM (2010). Tert-butylhydroquinone induces mitochondrial oxidative stress causing Nrf2 activation. Cell Biol Toxicol.

[25] Riethoven JJ (2010). Regulatory regions in DNA: promoters, enhancers, silencers, and insulators. Methods Mol Biol.

[26] Hayes JD, McMahon M (2009). NRF2 and KEAP1 mutations: permanent activation of an adaptive response in cancer. Trends Biochem Sci.

[27] Wakabayashi N, Slocum SL, Skoko JJ, Shin S, Kensler TW (2010). When NRF2 talks, who’s listening?. Antioxid Redox Signal.

[28] Stępkowski TM, Kruszewski MK (2011). Molecular cross-talk between the NRF2/KEAP1 signaling pathway, autophagy, and apoptosis. Free Radic Biol Med.

[29] Sriram N, Kalayarasan S, Sudhandiran G (2009). Epigallocatechin-3-gallate augments antioxidant activities and inhibits inflammation during bleomycin-induced experimental pulmonary fibrosis through Nrf2-Keap1 signaling. Pulm Pharmacol Ther.

[30] Liu YC, Hsieh CW, Wu CC, Wung BS (2007). Chalcone inhibits the activation of NF-kappaB and STAT3 in endothelial cells via endogenous electrophile. Life Sci.

[31] Pulkkinen KH, Yla-Herttuala S, Levonen AL (2011). Heme oxygenase 1 is induced by miR-155 via reduced BACH1 translation in endothelial cells. Free Radic Biol Med.

[32] Yu M, Li H, Liu Q, Liu F, Tang L, Li C, Yuan Y, Zhan Y, Xu W, Li W, Chen H, Ge C, Wang J, Yang X (2011). Nuclear factor p65 interacts with Keap1 to repress the Nrf2-ARE pathway. Cell Signal.

[33] Li Y, Jaiswal AK (1992). Regulation of human NAD(P)H:quinone oxidoreductase gene. Role of AP1 binding site contained within human antioxidant response element. J Biol Chem.

[34] Venugopal R, Jaiswal AK (1996). Nrf1 and Nrf2 positively and c-Fos and Fra1 negatively regulate the human antioxidant response element-mediated expression of NAD(P)H:quinone oxidoreductase1 gene. Proc Natl Acad Sci USA.

